# Incorporating Latent Variables Using Nonnegative Matrix Factorization Improves Risk Stratification in Brugada Syndrome

**DOI:** 10.1161/JAHA.119.012714

**Published:** 2020-11-10

**Authors:** Gary Tse, Jiandong Zhou, Sharen Lee, Tong Liu, George Bazoukis, Panagiotis Mililis, Ian C. K. Wong, Cheng Chen, Yunlong Xia, Tsukasa Kamakura, Takeshi Aiba, Kengo Kusano, Qingpeng Zhang, Konstantinos P. Letsas

**Affiliations:** ^1^ Tianjin Key Laboratory of Ionic‐Molecular Function of Cardiovascular disease Department of Cardiology Tianjin Institute of Cardiology Second Hospital of Tianjin Medical University Tianjin P.R. China; ^2^ Department of Cardiology The First Affiliated Hospital of Dalian Medical University Dalian China; ^3^ School of Data Science City University of Hong Kong Hong Kong Hong Kong SAR People’s Republic of China; ^4^ Laboratory of Cardiovascular Physiology Chinese University Shenzhen Institute Shenzhen P.R. China; ^5^ Second Department of Cardiology Laboratory of Cardiac Electrophysiology Evangelismos General Hospital of Athens Athens Greece; ^6^ School of Pharmacy University College London London UK; ^7^ Department of Pharmacology and Pharmacy University of Hong Kong Pokfulam Hong Kong; ^8^ National Cerebral and Cardiovascular Center Osaka Japan

**Keywords:** Brugada syndrome, depolarization, ECG, latent variable, nonnegative matrix factorization, repolarization, risk stratification, Arrhythmias, Electrophysiology, Sudden Cardiac Death

## Abstract

**Background:**

A combination of clinical and electrocardiographic risk factors is used for risk stratification in Brugada syndrome. In this study, we tested the hypothesis that the incorporation of latent variables between variables using nonnegative matrix factorization can improve risk stratification compared with logistic regression.

**Methods and Results:**

This was a retrospective cohort study of patients presented with Brugada electrocardiographic patterns between 2000 and 2016 from Hong Kong, China. The primary outcome was spontaneous ventricular tachycardia/ventricular fibrillation. The external validation cohort included patients from 3 countries. A total of 149 patients with Brugada syndrome (84% males, median age of presentation 50 [38–61] years) were included. Compared with the nonarrhythmic group (n=117, 79%), the spontaneous ventricular tachycardia/ ventricular fibrillation group (n=32, 21%) were more likely to suffer from syncope (69% versus 37%, *P*=0.001) and atrial fibrillation (16% versus 4%, *P*=0.023) as well as displayed longer QTc intervals (424 [399–449] versus 408 [386–425]; *P*=0.020). No difference in QRS interval was observed (108 [98–114] versus 102 [95–110], *P*=0.104). Logistic regression found that syncope (odds ratio, 3.79; 95% CI, 1.64–8.74; *P*=0.002), atrial fibrillation (odds ratio, 4.15; 95% CI, 1.12–15.36; *P*=0.033), QRS duration (odds ratio, 1.03; 95% CI, 1.002–1.06; *P*=0.037) and QTc interval (odds ratio, 1.02; 95% CI, 1.01–1.03; *P*=0.009) were significant predictors of spontaneous ventricular tachycardia/ventricular fibrillation. Increasing the number of latent variables of these electrocardiographic indices incorporated from n=0 (logistic regression) to n=6 by nonnegative matrix factorization improved the area under the curve of the receiving operating characteristics curve from 0.71 to 0.80. The model improves area under the curve of external validation cohort (n=227) from 0.64 to 0.71.

**Conclusions:**

Nonnegative matrix factorization improves the predictive performance of arrhythmic outcomes by extracting latent features between different variables.

**Nonstandard Abbreviations and Acronyms**
BrS: Brugada syndrome
NMF: nonnegative matrix factorization
SCD: sudden cardiac death

**Clinical Perspective**

**What Is New?**

- Nonnegative matrix factorization was used to extract latent features between clinical variables (initial type 1 pattern, syncope, atrial fibrillation) and electrocardiographic variables (QRS duration, QTc interval) in Brugada syndrome.
- Application of nonnegative matrix factorization improved prediction of incident ventricular tachycardia/ventricular fibrillation compared with logistic regression.

**What Are the Clinical Implications?**

- Incorporation of information on inherent higher order interrelations among variables improved prediction of ventricular tachycardia/ventricular fibrillation in Brugada syndrome.

Brugada syndrome (BrS) is an arrhythmogenic entity characterized by the high propensity of ventricular tachycardia/ventricular fibrillation (VT/VF) or sudden cardiac death (SCD).^1^, ^2^, ^3^ Subjects with spontaneous type 1 electrocardiographic (ECG) pattern and aborted SCD or syncope of arrhythmic origin are at the highest risk for future arrhythmic events and are advised to receive an implantable cardioverter‐defibrillator.^4^ However, emerging evidence clearly underscores our inability to stratify patients with BrS, particularly those who are asymptomatic. In a study of 50 individuals with SCD, the majority of SCDs related to BrS occurred in asymptomatic individuals (72%).^5^ In the SABRUS (Survey on Arrhythmic Events in Brugada Syndrome) registry, only 75% of patients who exhibited an arrhythmic event after receiving a prophylactic implantable cardioverter‐defibrillator complied with the 2013 class II indications of the Heart Rhythm Society, European Heart Rhythm Association, and Asia Pacific Heart Rhythm Society (syncope or inducible arrhythmias during programmed ventricular stimulation),^6^ suggesting that efforts are still required for improving risk stratification.

Although the BrS was initially described as a primary electrical disease,^2^, ^3^ recent evidence has demonstrated the presence of structural abnormalities mainly located at the epicardium of the right ventricular outflow tract.^7^, ^8^ Two principal mechanisms have been proposed to explain the pathophysiologic basis of BrS: the depolarization hypothesis, based on the slow conduction of the right ventricular outflow tract, and the repolarization theory related to the transmural dispersion of the right ventricular action potential morphology, driven by the loss of the spike and dome action potential in the right ventricular epicardium.^9^ So far, both repolarization and depolarization abnormalities have been associated with the development of VF in patients with BrS.^10^

Different risk stratification models incorporating clinical risk factors and ECG variables have been developed. However, they do not assess the interrelations between risk variables. In this study, we used nonnegative matrix factorization (NMF) to extract latent variables capturing the inherent interrelations among risk variables. NMF is a group of algorithms in multivariate analysis and linear algebra where a matrix **V** is factorized usually into 2 matrices **W** and **H**, with the property that all 3 matrices have no negative elements. This nonnegativity makes the resulting matrices easier to inspect. This enabled us to test the hypothesis that incorporation of latent variables can improve outcome prediction compared with logistic regression alone.

## Methods

### Inclusion of Study Subjects

This retrospective study received ethics approval from The Joint Chinese University of Hong Kong – New Territories East Cluster Clinical Research Ethics Committee (NTEC‐CUHK) and the Medical Ethical Review Committee of the Evangelismos General Hospital of Athens. Data on patients with BrS with spontaneous or drug‐induced (ajmaline 1 mg/kg or flecainide 2 mg/kg) type 1 BrS ECG pattern from Hong Kong, China, were retrospectively analyzed. The analyses for our cohort (the training set) were validated against an external cohort (the validation set) of data on patients with BrS from Athens, Greece; Dalian, China; Guangzhou, China; and Osaka, Japan. The anonymized databases have been made available by the investigators at Zenodo: https://zenodo.org/record/3266179; https://zenodo.org/record/3465811.^11^, ^12^, ^13^

The ECG diagnosis of BrS was strictly based on the recommendations of the 2015 European Society of Cardiology guidelines for the management of patients with ventricular arrhythmias and the prevention of SCD.^4^ The presence of tructural heart disease was excluded in all subjects. The following demographic and clinical details were extracted from medical case records: age, sex, aborted SCD, syncopal symptoms, spontaneous VT/VF, and inducible VT/VF during programmed ventricular stimulation. Programmed right ventricular apex stimulation was performed at 3 running cycle lengths (600, 500, and 430 milliseconds) with up to triple extrastimuli (minimum coupled extrastimuli of 200 milliseconds). Inducible ventricular arrhythmia was defined as any ventricular arrhythmia (VT/VF) causing syncope/circulatory collapse or requiring intervention for its termination.

### Electrocardiographic Variables

The 12‐lead ECGs were recorded at a paper speed of 25 mm/s with an amplification of 10 mm/mV (sampling rate: 10 seconds of ECG at 500 Hz, filters: 0.5–100 Hz). The following automated measurements were extracted from the baseline ECG records: heart rate, PR interval, QRS duration reflecting total depolarization time (beginning of Q to the end of S), and QTc interval reflecting total repolarization time with and without correction for heart rate using Bazett’s formula.

### Statistical Analysis

Data were expressed as median [interquartile range]. Differences between groups were tested using Kruskal‐Wallis analysis of 1‐way variance (ANOVA). The optimal cutoff values of the ECG variables, defined as the value maximizing the sum of sensitivity and specificity, for spontaneous VT/VF, were determined using the Youden index from receiver operating characteristic curve analysis. A *P* value <0.05 was considered statistically significant. Their predictive values were represented by the area under the curve (AUC) values from receiver operating characteristic analyses. Logistic regression was performed to determine the predictive value of different variables for spontaneous VT/VF. The results were presented as odds ratio (OR) with 95% CIs, with *P* values <0.05 considered statistically significant.

### Nonnegative Matrix Factorization

An NMF approach was used to capture inherent interrelations among risk variables. We then used the latent variables to develop a machine learning model to enhance the performance in predicting spontaneous ventricular tachycardia/ventricular fibrillation. First, we constructed matrix **V** representing the interrelations between clinical variables (initial type 1 pattern, syncope, atrial fibrillation [AF]) and ECG variables (QRS duration, QTc interval). Second, nonnegative matrix factorization was used to decompose matrix **V** into a core matrix **W** multiplied by a matrix **H** with different component cases (ie, number of latent variables generated). The generated latent variables were then combined with the risk variables as the input for logistic regression.

## Results

### Clinical Characteristics

This study cohort consisted of 149 patients with BrS from Hong Kong, China. The baseline demographic and clinical characteristics are presented in Table 1. The mean age was 50 (38–61) years old and 84% of the subjects were male. Syncope occurred in 65 (44%) and spontaneous VT/VF occurred in 32 (21%) patients. Atrial fibrillation was found in 10 (7%) patients. An initial spontaneous type 1 ECG pattern was recorded in 79 patients (53%). An implantable cardioverter‐defibrillator was inserted in 47 (32%) subjects. In the cohort, 44 (30%) underwent electrophysiological studies and 28 (64%) were positive tests.

**Table 1:** Demographic and Clinical Characteristics of Patients with Brugada Syndrome From Hong Kong Included in this Study (n=149)

Characteristics	Spontaneous VT/VF Group (n=32)	No Spontaneous VT/VF (n=117)	*P* Value
Male	30 (94)	95 (81)	0.087
Age at presentation (y)*	49 (35–68)	50 (39–59)	0.857
Implantable cardioverter‐defibrillator insertion	24 (75)	23 (20)	<0.0001†
Syncope	22 (69)	43 (37)	0.001†
Initial spontaneous type 1 pattern	21 (66)	58 (50)	0.107
Atrial fibrillation	5 (16)	5 (4)	0.023†
PVS performed	13 (41)	31 (26)	0.121
Positive PVS test‡	10 (77)	18 (58)	0.235
QRS	108 (98–114)	102 (95–110)	0.104
QT_c_	422 (399–449)	408 (386–425)	0.020†

High‐risk and low‐risk subjects were those with and without spontaneous VT/VF, respectively. Data were presented as number (%). *P* values were obtained from Fisher’s exact test (for frequency data) or Wilcoxon rank‐sum (Mann‐Whitney) test (for continuous data). PVS indicates programmed ventricular stimulation; and VT/VF, ventricular tachycardia/ventricular fibrillation.

a Denotes median (lower quartile to upper quartile).

b P<0.05.

c For PVS: this was carried out in 5 subjects in the high‐risk group and 104 subjects in the low‐risk group.

### Significant Predictors of Spontaneous VT/VF Using Logistic Regression

Compared with the nonarrhythmic group (n=117, 79%), the spontaneous VT/VF group (n=32, 21%) were more likely to suffer from syncope (69% versus 37%, *P*=0.001) and AF (16% versus 4%, *P*=0.023), as well as displayed longer QTc intervals (424 [399–449] versus 408 [386–425]; *P*=0.020). No difference in QRS interval was observed (108 [98–114] versus 102 [95–110], *P*=0.104). The results of logistic regression are shown in in Table 2. Age, sex, or initial spontaneous type 1 Brugada pattern did not significantly predict spontaneous VT/VF. By contrast, syncope (OR, 3.79; 95% CI, 1.64–8.74; *P*=0.002), AF (OR, 4.15; 95% CI, 1.12–15.36; *P*=0.033), QRS duration (OR, 1.03; 95% CI, 1.002–1.06; *P*=0.037), and QTc interval (OR, 1.02; 95% CI, 1.01–1.03; *P*=0.009) were significant predictors of spontaneous VT/VF. The receiver operating characteristic curves for the different variables described previously with the AUC values are shown in Figure S1A to D and Table 3.

**Table 2:** Univariate Logistic Regression for Spontaneous Ventricular Tachycardia/Ventricular Fibrillation

Parameter	Odds Ratio (T) (95% CI)	*P* Value	Odds Ratio (V) (95% CI)	*P* Value
Age at presentation (y)	1.00 (0.98–1.03)	0.918	1.00 (0.96–1.03)	0.822
Sex	0.29 (0.06–1.30)	0.105	1.71 (0.53–5.54)	0.368
Syncope	3.79 (1.64–8.74)	0.002*	9.09 (3.33–24.80)	<0.0001*
Initial spontaneous type 1 pattern	1.94 (0.86–4.39)	0.110	1.20 (0.47–3.07)	0.704
Atrial fibrillation	4.15 (1.12–15.36)	0.033*	2.50 (0.83–7.52)	0.103
Positive programmed ventricular stimulation test	2.41 (0.55–10.52)	0.243	0.27 (0.13–5.77)	0.404
QRS	1.03 (1.002–1.06)	0.037*	1.03 (1.00–1.06)	0.051
QT_c_	1.02 (1.004–1.03)	0.009*	1.02 (0.99–1.04)	0.150

left: training cohort from Hong Kong, China (T, n=149), right: validation cohort from Athens, Greece; Dalian, China; Guangzhou, China; and Osaka, Japan. (V, n=227).

a P<0.05.

**Table 3:** AUC of Continuous Variables for Predicting Spontaneous Ventricular Tachycardia/Ventricular Fibrillation

Parameter	Cutoff	AUC (95% CI)	Cutoff	AUC (95% CI)
Age at presentation (y)	63.5	0.51 (0.38–0.64)	43.5	0.48 (0.35–0.62)
QRS	107.5	0.59 (0.48–0.71)	110.5	0.68 (0.45–0.90)
QT_c_	433.5	0.63 (0.51–0.76)	409.5	0.60 (0.38–0.82)

left: training cohort (T), right: validation cohort (V). AUC indicates area under the curve.

### Regression Analysis With Latent Variables Extracted by NMF

Next, we constructed a variable matrix and then generated latent variables by performing NMF on the variable matrix to predict spontaneous VT/VF. The total number of components is denoted *d*, which indicates the number of extracted latent variables. We then combined the *d* additional latent variables and the 4 ECG variables in the 4‐point score system and then use them to fit a logistic regression. The regression performance under different combination cases of *d* latent variables are given in Table 4 and were compared with the performance with the baseline model (ie, logistic regression without latent variables). The AUC and 3 common metrics, precision, recall and F1 score, were reported to evaluate the performance. A 2‐fold cross‐validation was adopted to avoid overfitting concerns.

**Table 4:** Incorporation of Latent Variables and Its Effects on the AUC for Spontaneous Ventricular Tachycardia/Ventricular Fibrillation

# of Latent Variables (T)	Precision	Recall	F1	AUC	# of Latent Variables (V)	Precision	Recall	F1	AUC
Benchmark using logistic regression (*d*=0)	0.6901	0.7050	0.6975	0.7101	Benchmark using logistic regression (*d*=0)	0.5983	0.6131	0.6056	0.6383
*d*=2	0.7117	0.7265	0.7190	0.7187	*d*=2	0.6567	0.6552	0.6559	0.6759
*d*=3	0.7278	0.7341	0.7309	0.7269	*d*=3	0.6984	0.6567	0.6769	0.6809
*d*=4*	0.7749*	0.7828*	0.7788*	0.7965*	*d*=4	0.7048	0.6899	0.6973	0.6993
*d*=5	0.7719	0.7746	0.7732	0.7892	*d*=5	0.7139*	0.6960*	0.7048*	0.7092*
*d*=6	0.7806	0.7181	0.7480	0.7319	*d*=6	0.7123	0.6738	0.6925	0.6856

left: training cohort (T), right: validation cohort (V). AUC indicates area under the curve.

a P<0.05.

Using logistic regression as a baseline model, the AUC was 0.7101 (Table 4, left side). However, incorporating *d*=2, 3, 4, 5, and 6 additional latent variables by NMF led to improvement in the AUC values to 0.72, 0.73, 0.80, 0.79, and 0.73, respectively. Therefore, NMF improved the prediction performance over the baseline model using simple logistic regression with latent variables. The model achieved the best performance with *d*=4 latent variables. Our models were also validated using an external cohort from Athens, Greece; Dalian, China; Guangzhou, China; and Osaka, Japan (Table 4, right side). For the external cohort, the AUC was 0.64 using logistic regression, which was improved by NMF to 0.68, 0.68, 0.70, 0.71, and 0.69, through incorporating *d*=2, 3, 4, 5, and 6 additional latent variables, respectively. The model achieved the best performance with *d*=5 latent variables for external cohort validation.

## Discussion

The main findings of this study are that:

1. Syncope, AF, QRS duration, and QTc interval were significant predictors of spontaneous VT/VF;
2. Incorporation of information on inherent higher order interrelations among variables improved prediction of VT/VF.

The genesis of BrS remains controversial. Both depolarization (conduction delay within the right ventricular outflow tract, prolonged and fractionated potentials) and repolarization (imbalance of epicardial/endocardial repolarizing currents) abnormalities have been suggested to play crucial roles in the arrhythmogenesis of BrS.^14^, ^15^, ^16^ Previous studies have addressed the prognostic significance of specific depolarization and repolarization ECG markers. A prolonged QRS duration has been associated with an increased risk for arrhythmic events.^17^ For depolarization markers, QRS fragmentation has been associated with a 3.9‐fold increase in the risk of future arrhythmic events^18^ and a worse prognosis^19^ in BrS. The presence of a wide and/or large S‐wave in lead I has been suggested as a powerful predictor of ventricular arrhythmias. The presence of a wide S‐wave in lead I is possibly related to the delayed activation in the right ventricular outflow tract.^20^ Among the repolarization ECG markers, a prolonged QTc interval, which reflects delayed cellular repolarization, has been associated with an increased risk for VT/VF and SCD in BrS.^21^ Other significant ECG predictors include the presence of an early repolarization pattern,^22^ a low‐voltage type 1 ECG.^23^ AF^24^ and syncope^25^, ^26^ are observed in patients with BrS and are predictive of spontaneous VT/VF,^27^, ^28^, ^29^ as also seen in our study.

In this study, we used a method called NMF to extract latent variables and utilize this information for risk stratification. This led to significant improvements in the regression performance over the benchmark logistic regression‐based model. Only several groups have applied the NMF technique to ECG signal analysis. First, Yao et al reported that sparse constrained NMF improved classification of ECGs between the following diseases of right bundle branch block, left bundle branch block, premature ventricular contractions, paced rhythms, and patients without these abnormalities, using data from the Massachusetts Institute of Technology‐Beth Israel Hospital database.^30^ Second, Guyot et al used NMF for the preprocessing of long‐term ECGs, demonstrating that this simultaneously performed 3 tasks of denoising, baseline wander removal, and peak R detection.^31^ Here, we extend NMF analyses for the first time in patients with BrS , demonstrating that it can be used to improve classification accuracy of novel electrocardiographic depolarization‐repolarization indices between spontaneous VT/VF and nonarrhythmic groups.

### Limitations

Several limitations of this study should be recognized. First, this is a retrospective study and is therefore susceptible to bias inherent in this type of studies. Second, we fully recognize that the proposed electrocardiographic indices were measured at baseline and do not reflect the full dynamic nature of the electrophysiological changes in BrS. Indeed, improvement in risk stratification can be achieved with markers such as restitution indices or markers measured during stress^32^, ^33^, ^34^ or by assessing temporal variability of different ECG markers.^35^ Finally, the sample size was moderate with 376 patients, but our findings need to be further validated in larger prospective studies.

## Conclusions

The present findings suggest that nonnegative matrix factorization improves the predictive performance by extracting latent features between different variables.

## Sources of Funding

None.

## Disclosures

None.

## Supporting information


**Figure S1**
