# Supplementary material for: Incorporating Latent Variables Using Nonnegative Matrix Factorization Improves Risk Stratification in Brugada Syndrome
Source: J Am Heart Assoc. 2020 Nov 10;9(22):e012714. doi: 10.1161/JAHA.119.012714 (PMC7763720; doi:10.1161/JAHA.119.012714)

SUPPLEMENTAL MATERIAL

Figure S1. Receiver operating characteristic curves for syncope (A), atrial fibrillation (B), QTc interval (C) and QRS duration (D).

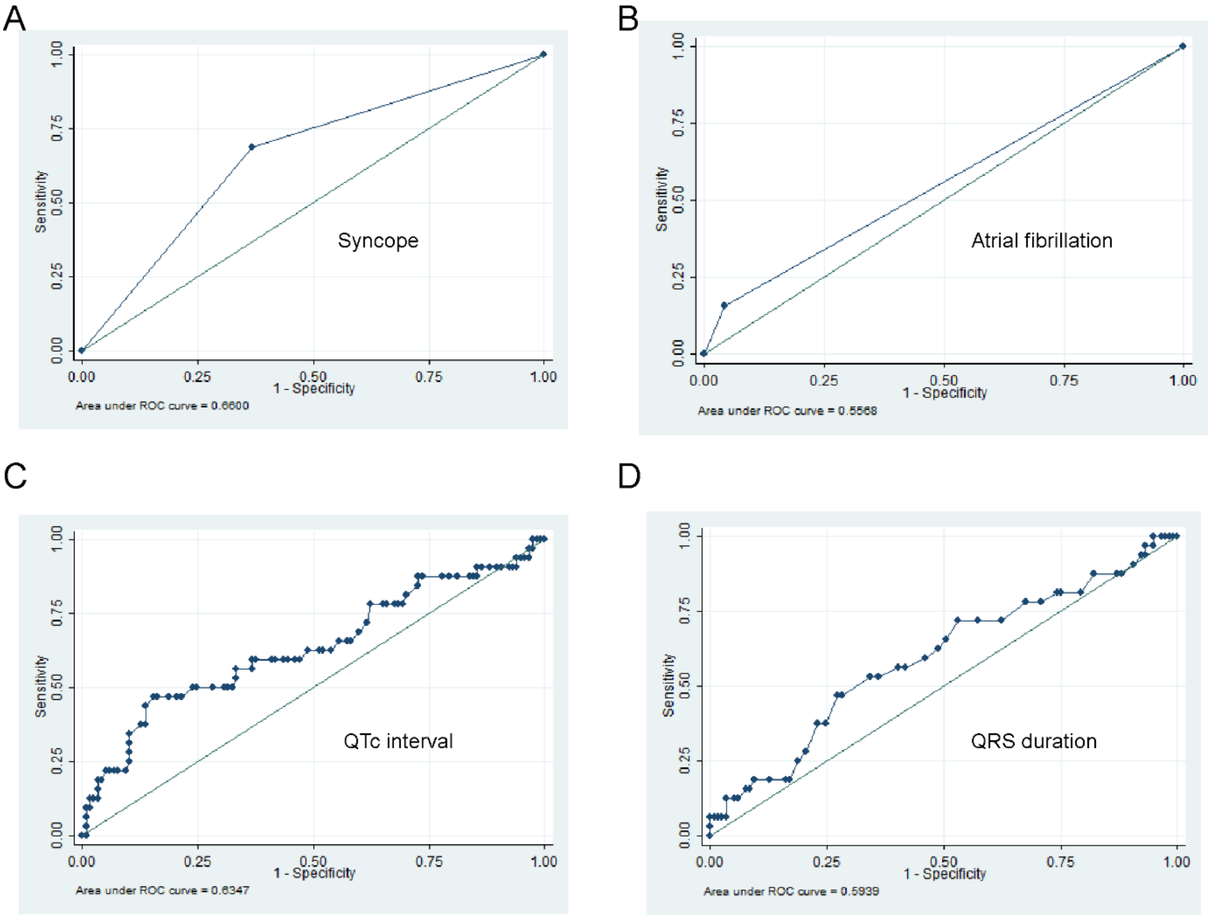

Supplement: Supplementary file 1 — Figure S1 [file JAH3-9-e012714-s001.pdf]
